# Effects of Raw and Roasted Cocoa Bean Extracts Supplementation on Intestinal Enzyme Activity, Biochemical Parameters, and Antioxidant Status in Rats Fed a High-Fat Diet

**DOI:** 10.3390/nu12040889

**Published:** 2020-03-25

**Authors:** Dorota Żyżelewicz, Joanna Oracz, Małgorzata Bojczuk, Grażyna Budryn, Adam Jurgoński, Jerzy Juśkiewicz, Zenon Zduńczyk

**Affiliations:** 1 Institute of Food Technology and Analysis, Faculty of Biotechnology and Food Sciences, Lodz University of Technology, 90-924 Lodz, Poland; joanna.oracz@p.lodz.pl (J.O.); malgorzata.bojczuk@gmail.com (M.B.); grazyna.budryn@p.lodz.pl (G.B.); 2 Department of Biological Functions of Food, Institute of Animal Reproduction and Food Research, Polish Academy of Sciences, 10-748 Olsztyn, Poland; a.jurgonski@pan.olsztyn.pl (A.J.); j.juskiewicz@pan.olsztyn.pl (J.J.); z.zdunczyk@pan.olsztyn.pl (Z.Z.)

**Keywords:** *Theobroma cacao* L., cocoa beans extracts, intestinal microbiota activity, antioxidant parameters, oxidative stress markers, lipid profile, metabolic dysfunction

## Abstract

The aim of the study was to analyze the influence of diet containing the polyphenol-rich material on intestinal enzyme activity, oxidative stress markers, lipid metabolism and antioxidant status of laboratory rats. The animals were fed high-fat diet supplemented with freeze-dried water extracts of raw and roasted cocoa beans of *Forastero* variety. The observed changes indicated the biological activity of polyphenols and other components of the prepared cocoa beans extracts (CBEs). The presence of raw and roasted CBEs in the diets diversified the activity of the enzymes of the cecal microflora of rats. Both CBEs beneficially affect the antioxidant status of the serum, even in relation to the control standard group. The experimental cocoa bean preparations showed no significant effect on the mass of rats’ liver, heart, and kidneys, but varied some parameters of the antioxidant status of their organisms. The raw CBE in rats fed with the high-fat diet shows a high ability to inhibit lipid peroxidation in heart and more effectively increases hepatic reduced glutathione (GSH) concentrations compared to the roasted CBE, which did not show any significant effect. Moreover, supplementation with both CBEs significantly affects the volatile fatty acids concentration in the rats’ cecum. Results of this study contribute to the evidence that dietary supplementation with raw and roasted CBEs can exert health-promoting effects, however further studies are necessary.

## 1. Introduction

Plants constitute one of the most significant sources of phenolic compounds known mostly for their antioxidant properties [1]. Cocoa beans and their co-products are also rich in phenolic compounds, especially flavonoids [2,3]. Although ancient civilizations recognized cocoa beans as a health-promoting raw material, cocoa-derived products have only recently gained recognition for use as a significant source of functional ingredients in foods, nutraceuticals, or cosmetics [4]. These beneficial properties, attributed mainly to the presence of a significant number of bioactive compounds, are a perennial topic of investigation [1,5,6]. Cocoa beans due to the presence of polyphenols exhibit diverse biological activities, such as antioxidant, antiradical, antimicrobial, anti-inflammatory, antithrombotic, antihypertensive, anticarcinogenic, antiallergic, immunomodulatory, and cardioprotective, resulting in the protection against diseases such as cardiovascular diseases, cancer or neurodegenerative disorders among others [7,8,9,10,11,12,13,14,15]. Cocoa flavan-3-ols, mostly (+)-catechin and (-)-epicatechin, are easily absorbed by the intestinal walls and brought to the bloodstream after being rapidly and extensively metabolized into numerous structurally related metabolites, thus presenting a great potential for action in the human organism [7,16] On the other hand cocoa beans mainly contains polymeric forms of flavan-3-ols that are poorly absorbed in the small intestine, and the majority of those compounds reach the colon where are extensively metabolized by colon microflora into a wide range of low molecular weight phenolic acids [7,16].

The content of phenolic compounds in cocoa beans differs depending on their variety, the growing region, harvesting practices, and processing steps. After the cocoa beans have been released from pods, they are subjected to a number of pre-processing steps (fermentation and drying) to obtain high quality of final products. The process of roasting is, however, the most important in terms of flavor formation. Reactions occurring in cocoa beans during the roasting depend on many factors, for example, the chemical composition of the beans, roasting temperature and time, or relative humidity of the roasting air [17,18,19,20]. During roasting, the acidity of beans is decreased due to the reduction of the volatile acids’ concentration. Unroasted cocoa beans, therefore, are bitter, acidic, astringent, and nutty in flavor [21]. The process of roasting leads to the formation of the early, advanced and final Maillard reaction products (MRPs) in the beans. These compounds influence such parameters of roasted products as taste, color, aroma, and texture, and, what is more, melanoidins formed at the advanced or final stage of the Maillard reactions are characterized by a strong antioxidant and antimicrobial activities as well as antihypertensive properties [22,23,24]. Moreover, cocoa beans processing, including fermentation, drying and roasting leads to a significant change in the phenolic compounds content, and thus the biological activity of cocoa derived products [3,19,20].

As it is known, the unbalanced diet as well as genetics and environment are key factors responsible for the development of obesity and easily flamed up. The first one, who introduced a description of so-called “high-fat’” diet was Masek and Fabry [25]. This type of diet contains an increased energy load from fat and therefore nowadays refers to the dietary habits of a significant part of the population of economically developed countries. Subsequent studies have shown a positive relationship between dietary fat intake and obesity [26,27]. Epidemiological studies have revealed the influence of high-fat diets on promoting the hyperglycemia and insulin resistance. The effect of high-fat diets on muscle and liver physiology as well as insulin signal transduction has aroused since then the curiosity of the scientists [28]. Animal rodent models are considered useful tools for studying dietary obesity as they readily gain weight when fed high-fat diets [29]. Previous studies of animals and humans have suggested that cocoa intake could be related to the decreased risk of cardiovascular diseases, diabetes, and obesity [30,31,32,33,34,35,36,37,38,39]. Our recent study also showed that raw and roasted cocoa beans extracts (CBEs) as well as purified monomeric flavan-3-ols fraction isolated from them differently affected the activity of cecal enzymes and the content of volatile fatty acids in the gut but would not significantly affect the hematological parameters of rats fed a high-fat and low-fiber diet [40].

In continuation with our previous study, this work was designed to evaluate the influence of diet enriched with the extracts from raw and roasted cocoa beans of *Forastero* variety on the intestinal enzyme activity, lipid peroxidation, antioxidant status, and lipid profile of laboratory rats fed a high-fat low-fiber diet. 

## 2. Materials and Methods 

### 2.1. Materials

The following study analyzed the influence of the extracts obtained from raw and roasted cocoa beans (*T. cacao* L.) of *Forastero* variety harvested in Peru, added to the diet of laboratory rats. Cocoa beans were purchased from Barry Callebaut Polska Sp. z o.o. (Lodz, Poland). Standards of catechin (C), epicatechin (EC), epigallocatechin (EGC), gallic acid (GA), procyanidin B2 (PC B2), procyanidin C1 (PC C1), quercetin (Q), quercetin 3-*O*-glucoside (Q-Glu), quercetin 3-*O*-arabinoside (Q-Ara), quercetin 3-*O*-galactoside (Q-Gal), caffeine (CF), theobromine (TE), acetonitrile of HPLC grade (≥99.9 %), and formic acid for LC–MS (~98%) were all purchased from Sigma-Aldrich (St. Louis, MO, USA). All other chemicals were of analytical grade and reagents were prepared according to standard analytical procedures. 

### 2.2. Roasting Experiments

Raw cocoa beans are the basic raw material for chocolate production. One of the stages of its preparation is roasting, which contributes to the loss of phenolic compounds and affects the biological properties of these beans. However, as a result, the Maillard reaction products including melanoidins are formed. These compounds have confirmed bioactive properties including strong antioxidant activity, anticancer, and antimicrobial properties. Melanoidins may also act as dietary fiber and promote the growth of beneficial like bifidobacteria in the gut [24,41] For this reason, extracts from roasted cocoa beans were also obtained during the research. 

Cocoa beans were roasted according to the method described by Żyżelewicz et al. [42]. The parameters of the roasting air were temperature: T = 135 °C; time: 35 min; relative air humidity: RH = 0.3%; flow velocity: v = 1.0 m/s. The roasting process was terminated when water content in the final product dropped to around 2%. These parameters were selected in order to preserve as many polyphenols as possible along with the minimal presence of compounds of negative nature in the roasted material like acrolein, acrylamide, and polycyclic aromatic hydrocarbons [18,20].

### 2.3. Preparation of Cocoa Bean Extracts

The CBEs were prepared from both raw and roasted cocoa beans according to the procedure described by Żyżelewicz et al. [43]. After dehulling, grinding and sieving raw and roasted cocoa beans were extracted with distilled water in a ratio of 1:3 (w/v). In the present study, we have selected water as a solvent due to the fact that the ultimate goal of our research is the application of CBEs in the food industry. The obtained ground cocoa kernels were extracted at 60 °C for 30 minutes using an SV 1422 Memmert (Schwabach, Germany) water bath shaker. Subsequently, the suspensions were filtered under vacuum using a vacuum pump KNF 18 035.3 N (Neuberger, NJ, USA). After this step, the obtained extracts were frozen at −20 °C, freeze-dried (−50 °C, 0.9 mPa) in a BETTA2-8LSC plus Christ freeze drier (Martin Christ Gefriertrocknungsanlagen GmbH, Osterode am Harz, Germany) and stored in plastic bags at −24 °C until further usage. 

### 2.4. UHPLC-DAD-ESI-MS/MS Analysis of Phenolic Compounds and Methylxanthines

Phenolic compounds and methylxanthines were identified and quantified in obtained CBEs according to the method described by Żyżelewicz et al. [43]. A UHPLC-DAD-ESI-MS/MS analyzes were performed using an UHPLC+Dionex UltiMate 3000 liquid chromatographic system equipped with a diode array detector with multiple-wavelength (Thermo Fisher Scientific Inc., Waltham, MA, USA) and an UHRQ-TOF-MS/MS (Bruker Daltonics GmbH, Bremen, Germany) using an electrospray ionization (ESI) source. Separation of phenolics and methylxanthines was carried out using a Accucore™ C18 2.6 μm, 150 mm × 3.0 mm i.d. column (Thermo Scientific, Waltham, PA, USA) thermostated at 30 °C. The mobile phases were eluent A, 0.1% formic acid in water (v/v), and eluent B, 0.1% formic acid in mixture of acetonitrile/water/ (80/20/, v/v). The flow rate was 0.3 mL/min and the gradient was as follows: 0–5 min, 5% B; 5–6 min, 5–8% B; 6–25 min, 8–15% B; 25–30 min, 15–20% B; 30–35 min, 20–25% B; 35–38 min, 25–30% B; 38–45 min, 30–85% B; 45–52 min, 85–5% B; 52–62 min, 5% B. UV-Vis detection was performed at 280 nm for gallic acid, monomeric flavan-3-ols, procyanidins and methylxanthines, and at 365 nm for quercetin and its derivatives. The sample injection volume was 10 μL. In the ESI-MS system, the drying gas temperature were 200 °C, and the positive and negative ion capillary voltage was set at 5.5 and −4.5 kV, respectively. Nitrogen was used as both drying gas (8.0 L/h) and nebulizing gas (1 bar). The collision energy was 20 eV. Full-scan MS and MS/MS spectral data were acquired over a range from m/z 50 to 1500. The MS/MS spectra were obtained in collision-induced dissociation (CID) mode using nitrogen as the collision gas. Instrument control, data acquisition, and evaluation were done with the OTOFControl 3.2, HyStar 3.2, and Chromeleon 6.8.1 chromatography Data System software, respectively. Identification of phenolic compounds and methylxanthines was based on the comparison of their retention times, UV-Vis absorbance spectra, full scan mass spectra, and MS/MS fragmentation patterns with those of corresponding standards analyzed under identical conditions. Quantification of individual phenolic compounds was carried out using external standard method as indicated in Żyżelewicz et al. [43]. The analysis of both CBEs by UHPLC-DAD-ESI-MS/MS allowed for the identification and quantification of a total eleven phenolic compounds and two methylxanthines (Table 1). The phenolic compounds and methylxanthine content were expressed as mg per g dry weight (mg/g DW). 

### 2.5. Animal Study

#### 2.5.1. General Information on the Tested Subject and Investigated Diets

The study was conducted in compliance with the European Guidelines for the Care and Use of Laboratory Animals (The ethic approval number: 71/2014), according to the proposal approved by the Local Institutional Animal Care and Use Committee (Olsztyn, Poland). The experiment was conducted on 28 male 6-wk (± 2 days) old Wistar laboratory rats of similar initial body weight (average BW 165.9 g). The animals were randomly divided into 4 groups, 7 rats each. The experiment lasted 4 weeks. During this time due to strict individual feed consumption recorded daily, the animals were kept separately in plastic cages (one rat per cage with transparent walls enabling eye contact with other animals) under the conditions of stable temperature (21–22 °C) and relative humidity of 50–70%, with a ventilation rate of 15 air changes per hour and 12-hour light/12-hour dark cycle. Throughout the whole experiment, the rats had free access to water and food. The experimental diets were a modification of semi-synthetic AIN-93G diet [44], developed by American Institute of Nutrition for rats during their intensive growth. The source of protein in the diet was casein, and the sources of minerals and vitamins were standard mineral and vitamin blends, respectively AIN-93G-MX and AIN-93G-VX [45]. In all diets, the DL-methionine was added in order to supplement the deficiency of this amino acid in casein. The detailed composition of all experimental diets is presented in Table 2. The rats were fed four types of diets, two control ones (D_CS_ and D_CF_) and two supplemented, one with freeze-dried raw (D_RW_) cocoa beans extract and the other one with freeze-dried roasted cocoa beans extract (D_RT_) [40]. D_CS_ diet was an example of a standard one, with a standard composition providing adequate levels of dietary fiber (5% cellulose) and appropriate energy ratio from fat (7% rapeseed oil) and easily digestible carbohydrates (10% sucrose and 53% corn starch). D_CF_ diet, on the other hand, was an example of a high-fat diet. The two supplemented diets were a modification of this “faulty” one. The aim was to check the possibility of limiting the negative effects of such type of diet on physiological indices of the tested animals.

The investigated diets were administered during the period of intensive growth of tested animals, i.e., age of 6 weeks, with feed intake controlled daily.

#### 2.5.2. Body Composition Analysis

The influence of the phenolic compounds present in the rats’ diet on such parameter as body fat and lean tissue was determined by time-domain nuclear magnetic resonance technique using a Minispec LF 90II analyzer (Bruker Optics, Bremen, Germany) as indicated in Żyżelewicz et al. [46]. The body lean and fat tissue mass for each animal were determined at weeks 0 (“initial” stage) and 4 (“final” stage) of the feeding experiment. 

#### 2.5.3. Sample Collection and Analysis

After the termination of the study, the rats were weighed and anesthetized with sodium pentobarbital (50 mg/kg body weight) according to Close et al. [47]. The samples of small intestine, caecum and colon, including digesta as well as tissue samples from rats’ organs (liver, kidneys, heart) were collected post-mortem. The small intestine, cecum, and colon with contents were weighed and pH of the content was determined using microelectrodes (pH-meter, model 301, Hanna Instruments, Amorim, Portugal). The activity of enzymes present in rats’ gastrointestinal tract such as α-glucosidase (maltase), *β*-fructosidase (sucrase), and *β*-galactosidase (lactase) was measured using a modified method of Dahlqvist [48]. The ammonia content in the cecum samples was determined according to Hofirek and Haas [49]. 

The profile of volatile fatty acids (VFAs) in the rats’ cecum content was determined using a gas chromatograph equipped with FID detector (Shimadzu GC-14A, Kyoto, Japan) as described in Żyżelewicz et al. [46].

The activity of glycolytic enzymes in the samples of rats’ cecum content was determined according to the method described by Djouzi and Andrieux [50] modified by Fotschki et al. [51] and Jarosławska et al. [52]. The activity of the enzyme (U) in 1 g of the cecum content was expressed in μmol *p*-(*o*-) nitrophenol released in 1 minute [40]. 

The blood was collected from the caudal vein. The serum was prepared by solidification and centrifugation at 305 × *g* for 15 min at 4 °C and stored at −70 °C until analyses. The biochemical indicators in the obtained serum such as the serum concentration of glucose, total cholesterol (TC), HDL cholesterol fraction (HDL-C) and uric acid were determined as described in Żyżelewicz et al. [46]. The atherogenic index (AI) was calculated based on the serum lipid profile, using the following formula: [(TC – HDL)/HDL] (Equation 1). The non-HDL fraction was calculated by subtracting HDL-C from total cholesterol value (Equation 2).

The serum antioxidant activity of water-soluble and lipid-soluble substances (ACW and ACL, respectively) was determined by means of photochemiluminescence detection method using the procedure described by Żyżelewicz et al. [46]. Ascorbate and Trolox calibration curves were used to evaluate ACW and ACL, respectively.

The concentration of reduced (GSH) and oxidized glutathione (GSSG) in liver tissue was determined using an enzymatic recycling assay developed by Rahman et al. [53] and expressed as μmoles of GSH or GSSG per gram of tissue. The content of thiobarbituric acid-reactive substances (TBARS) in the kidneys, liver, and heart was determined spectrophotometrically at 532 nm according to the procedure described by Botsoglou et al. [54] and expressed as ng of malondialdehyde per g of tissue.

### 2.6. Statistical Analysis

All experiments were performed in triplicate. The results of all determinations are presented as mean ± standard error (SEM). The obtained data were subjected to statistical analysis using STATISTICA 10 software (StatSoft Inc., Tulsa, OK, USA). The determination comprised of both, average values and one-way analysis of variance ANOVA Tukey’s honest significant difference post hoc test. For all statistical analysis, *p* < 0.05 was considered as statistical significance.

## 3. Results and Discussion

Increasing the energy value in a high-fat diet resulted in lower feed intake compared to the standard diet (D_CS_) (Table 3). The differences in feed and energy intake in control D_CS_ group compared to the three high-fat groups did not significantly affect the body weight gain (BWG) of rats. 

However, the groups fed the high-fat diets with the supplementation of 2% raw and roasted CBEs (D_RW_ and D_RT_, respectively) in place of the 2% corn starch were characterized by slightly lower weight gain than after administration of the control high-fat diet (D_CF_). Moreover, the intake of high-fat diets significantly increased the absolute amount of lipid fraction in the body of rats, compared to the standard control diet, whereas the relative fat content, related to the body weight of the animals, was similar in all experimental groups. This finding was consistent with results of Min et al. [55] who reported that cocoa polyphenol extract administration at a high dose (200 mg/g BW) in a high-fat diet significantly reduced body weight gain in obese C57BL/6N mice while at the lowest dose (40 mg/g BW) the anti-obesity effect was no significant. Slightly different results were also revealed by Aranaz et al. [35] who studied the influence of an ethanolic complete cocoa extract supplementation on body weight gain, food efficiency, adiposity accumulation, liver steatosis, glucose homeostasis and lipid metabolism in high fat/high sucrose diet induced obese Wistar rats. These authors demonstrated that after 10 weeks, supplementation of a high fat/high sucrose diet with a cocoa extract significantly reduced the body weight gain and food efficiency compared with the non-supplemented high fat/high sucrose group. These differential effects could have resulted from many factors such as difference in the composition of cocoa extract (i.e., phenolic compounds, methylxanthines, fiber), dose of the extracts and the length of the experiment [35,55]. Several mechanisms have been proposed whereby cocoa intake could affect body weight gain in a high-fat diet [55,56]. One explanation is that cocoa flavonoids can inhibit fat digestion, absorption, and deposition [55,56]. Another possible mechanism is interaction between phenolic compounds and the gut microbiota [55]. Furthermore, it was established that consumption of methylxanthines accelerates cellular lipolysis and stimulates thermogenesis, which in consequence leads to a decrease in body mass [57].

Table 4 presents results obtained for the analyzed parameters of rats’ small intestine, cecum, and colon. There were no significant differences in the weights and pH of the small intestine between all tested groups. All three high-fat diets (control and supplemented) lowered the activity of the small intestine mucosa enzymes, as compared to the D_CS_ one. The supplementation of cocoa beans extracts had statistically significant effect on the saccharase activity but did have a significant effect on maltase and lactase activity compared to the D_CF_ diet.

The rats’ cecum mass and content were also not significantly differentiated between the groups. This indicates that the extracts introduced into the diet did not have a significant impact on the transit of the digestive tract in the gut. In the case of the D_RW_ and D_RT_ groups, an increased hydration of the cecum content was observed, and the amount of colonic content has significantly increased. The percentage water content of cecum and the colon content were higher in the D_RW_ diet group than in the D_RT_ diet group. This may be due to the difference in the bioactive compounds’ composition and content of CBEs. The raw CBE contained more polymeric flavonoids and soluble fiber. These findings are consistent with previous reports of Juśkiewicz et al. [58], indicating that an increase in the hydration of cecal digesta may result from a proliferation of bacterial strains and water binding by osmoactive substances. Dietary fiber increases hydration of digesta residue in the cecum. It has been reported that polymeric flavonoids and fiber reach the colon where they are fermented by the colonic microbiota to low molecular weight metabolites that can affect the gut environment [56,57]. On the other hand, Massot-Cladera et al [56] investigated the impact of cocoa, cocoa fiber, or inulin on the gut microbiota composition in Wistar rats. It was reported that phenolic compounds and other substances, like theobromine found in whole cocoa, may influence the fermentation of the fiber through both anti-bacterial and prebiotic actions against gut bacteria [30,56,57]. Thus, we can conclude that replacing cellulose with CBEs that contained phenolic compounds and methylxanthines may affect bacteria colonizing the last part of the digestive tract and may have a trophic effect on the cecum and colon of rats [59]. The mass of the colon wall was similar between all groups fed a high-fat diets with and without CBEs. The highest mass of the colon tissue was reported for the group fed the control standard diet contained high amounts of cellulose and corn starch. No influence of the supplementation with CBEs was reported on the pH value of intestinal contents derived from the following sections of the gastrointestinal tract of rats.

The applied experimental diets also resulted in a diversification in the concentration and total production (pool) of VFAs in the cecum content, including the concentration of the most important one, namely acetic acid (Table 5). 

The obtained results indicate an increased concentration of the most important VFAs (acetic, propionic, and butyric) in case of the diet supplemented with roasted CBE. In the case of both, propionic and butyric acids, these changes were significant (*p* < 0.05). These results may be ascribed to the lower content of phenolic compounds in roasted CBE, which could inhibit colonic fiber metabolism [30,56]. In comparison with D_CF_ diet, a favorable increase in the proportion of butyric acid in the sum of VFAs was found in the case of both supplemented diets. This result is very important because butyrate is one of the most important VFAs in human health. For example, butyric acid plays a key role in normal development of epithelial cells in the large intestine and can slow a colorectal cancer’s development [59,60]. This increase, however, was significantly important only in the case of the D_RT_ diet. Both supplemented diets reduced the concentration of two putrefactive acids, iso-butyric and iso-valeric, as compared to the control high-fat diet (D_CF_). This indicates that CBEs could significantly reduce the content of these VFAs, formed as a result of the amino acid degradation process in the cecum of rats. The D_RW_ diet resulted in lower or equal concentration of the third acid classified to that group, namely valeric acid, as compared to both control groups, D_CS_ and D_CF_, respectively. Similarly, to the results of this study Massot-Cladera et al. [56] reported that concentrations of butyric acid was significantly increased in the group supplemented with 10% cocoa compared with the control group. Martín-Peláez [57] also demonstrated that administration of a diet containing 10% cocoa or a diet including 0.25% theobromine resulted in an increase in the short chain fatty acids concentrations, as compared to a standard diet. The diet containing 10% cocoa enhanced butyric and acetic acid production [57].

The presence of raw and roasted CBEs in the diets differentiated the activity of the enzymes of the cecal microbiota of rats (Table 6). In all groups, similar total activities of α-glucosidase, as well as *β*-glucuronidase, were determined. However, in comparison to the control standard and control high-fat diets, both raw and roasted CBE supplementation caused the significant increase in *β*-glucosidase and α-galactosidase activity, while roasted CBE was also associated with greatest decreased activity of *β*-galactosidase. The α-glucosidase is an important glycolytic enzyme [61], while *β*-glucosidase is a key enzyme that is capable of specifically catalyze hydrolysis of cellulose and could also be involved in transformations of a range of different plant-derived glucosides into aglycones [62]. The released compounds might exhibit either harmful or health-promoting effects. Thus, the observed differences could be explained by variations in the contents of phytochemicals in the CBEs studied. For example, the total phenolic contents were found considerably higher in raw CBE but roasted CBE contains higher amounts of both methylxanthines and MRPs, especially high molecular weight melanoidin fractions [43]. A differentiated effect of diets on the release degree of the enzymes was observed. For instance, a significant difference was observed in the case of α-galactosidase enzyme between groups D_CS_, D_RT_ and D_CF_, D_RW_. D_RT_ diet also significantly increased the release degree of *β*-galactosidase, compared to all other groups. The obtained results are confirmed by literature data indicating that supplementation with polyphenolic-rich extracts advantageously modulates the enzymatic activity of the cecum microbiota [63,64]. 

Fotschki et al. [63,64] observed that the introduction to the diet of rats polyphenolic compounds belonging to the group of ellagotanins and flavan-3-ols, contributed to a significant reduction in β-glucuronidase activity, which is an indicator of adverse changes in the composition of the intestinal microflora, e.g., an increase in the population of pathogenic bacteria, including *Escherichia coli, Pseudomonas aeruginosa, Staphylococcus,* and *Clostridia* [54]. Moreover, this enzyme is responsible for the transformation of xenobiotics in the gastrointestinal tract into more toxic substances [61,65]. Increased levels of *β*-glucuronidase is an important indicator of the increased risk of cancer [66]. However, the results presented in our study demonstrated that diets supplemented with CBEs only slightly decreased the *β*-glucuronidase activity compared to both control standard and high-fat diets, but these tendencies were not significant (*p* > 0.05). This could be explained by the differences in the composition of phenolic compounds in tested plant materials and their absorption, bioavailability and metabolism [16,62]. Furthermore, the intake of different phenolic compounds could modulate the gut microbiota differently avoiding the growth of pathogenic bacteria and promote the growth of beneficial bacteria [62]. This possibility was also indicated in the study conducted by Álvarez-Cilleros et al. [30] where cocoa intake modified the gut bacterial composition towards a healthier profile of microbiota in diabetic rats [30]. The authors suggested that alterations in the gut microbiota improve glucose homeostasis and this phenomenon could be associated with the potential anti-obesity mechanisms of cocoa polyphenols include improvement glucose homeostasis and gut health [30,31]. 

**Figure 1:**
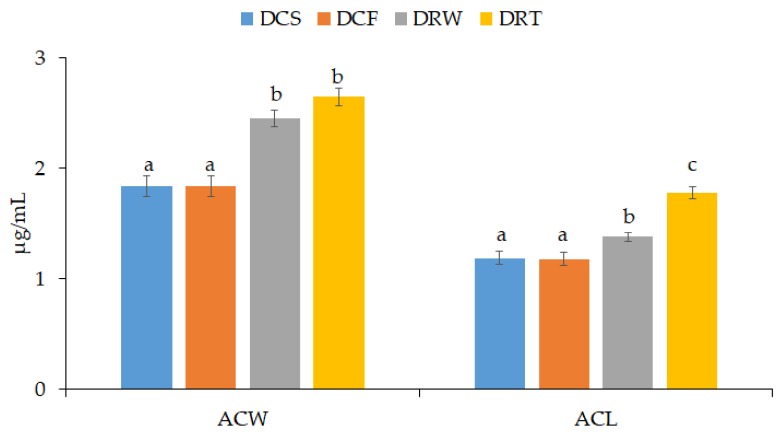
The antioxidant capacity of water-soluble and lipid-soluble substances in the serum. Diet: D_CS_—control standard diet, D_CF_—control high-fat diet, D_RW_—diet enriched with freeze-dried raw cocoa beans extract, D_RT_—diet enriched with freeze-dried roasted cocoa beans extract. Results are presented as means ± SEM from triplicate assays. Bars with the same lowercase letter (a–c) within antioxidant capacity derived from hydrophilic/lipophilic antioxidants do not differ significantly according to Tukey’s HSD test at *p* > 0.05. ACW—antioxidant capacity of water-soluble substances expressed as μg of ascorbic acid equivalents per mL, ACL—antioxidant capacity of lipid-soluble substances expressed as μg of Trolox equivalents per mL.

The experimental cocoa beans preparations showed no significant effect on the mass of rats’ liver, heart, and kidneys, but varied some parameters of the antioxidant status of their organisms (Figure 1, Table 7). The presence of both CBEs in the diets resulted in a significant increase in the antioxidant potential of the aqueous fraction (ACW) and lipid fraction (ACL) of the serum. The rats fed the high-fat diet with both CBEs had an ACW and ACL level significantly higher than the levels in high-fat diet animals but also higher than the levels in D_CS_ group (Figure 1). 

The much higher increase in ACW reported in the case of both supplemented diets indicated the antioxidant effect of mainly water-soluble components of cocoa beans. Interestingly, the stronger antioxidant potential was observed for roasted CBE, in comparison to the raw CBE which contained more polyphenolic compounds. The increase of antioxidant potential in rats fed roasted CBE may be ascribed to the presence of new antioxidant compounds including melanoidins formed during roasting [24,67]. Previous studies have shown that melanoidins have antioxidant properties mainly due to the presence of phenolic compounds, and especially phenolic acids, in their structure [24,68,69]. Phenolic compounds can be incorporated in the melanoidin structure during the roasting of cocoa beans through covalent and non-covalent interactions [70]. However, the bioavailability and stability of these compounds in the digestion and absorption process affect greatly their health benefits. Large part of phenolic groups associated with the fiber or melanoidin fractions are not fully released and absorbed in the small intestine and reach the colon where are metabolized by colon microbiota. The colonic catabolites of these compounds with low molecular weight can achieve higher concentrations in blood and reach the target organs in the body [7,16,71]. Thus, this result indicates that both raw and roasted CBEs may reduce the overall oxidative stress and the risk of associated degenerative disorders. In relation to the D_CF_ group, the supplementation with both CBEs, increased the concentration of the sum of glutathione in the rats’ liver, significantly reduced the pro-oxidative effect of the high-fat diet (Table 7). Both extracts increased the concentration of reduced glutathione (GSH) but did not increase the amount of oxidized glutathione (GSSG). 

As a result, the GSH/GSSG ratio in the D_RW_ and D_RT_ groups increased to the level of the control diet D_CS_. GSH/GSSG profile was the most beneficial for the diet with D_RW_ and increased 3.5-fold compared to D_CF_. GSH is considered as most important intracellular defense against reactive oxygen species (ROS) induced oxidative damage and its ratio with GSSG may be used as a marker of oxidative stress [72]. The level of TBARS was increased in high-fat groups compared with control group. Dietary supplementation of raw CBE significantly decreased the concentration of TBARS in heart of high-fat groups. Similar results were obtained in studies of other authors [64,65]. Many studies have shown that cocoa and cocoa products can exert strong antioxidant properties and improve resistance to oxidative stress [7]. It could be possible that the presence of phenolic compounds in the raw CBE may effectively prevent oxidative stress and inflammation in the rats organs, including heart, liver, and kidneys. 

As shown in Table 8, the addition of CBEs significantly influenced the glucose concentration in the blood serum. Both preparations caused an increase in blood glucose levels when compared with D_CS_ and D_CF_ groups. Higher blood glucose concentration in rats fed diets containing CBEs may result from the presence of soluble sugars in these preparations, extracted from cocoa beans. No significant tendencies (*p* > 0.05) were found in the other analyzed biochemical and enzymatic serum indicators between control groups and groups fed supplemented diets. However, the dietary treatments with cocoa beans preparations resulted in some beneficial changes in serum lipid profile in rats fed with a high-fat diet. In this study, TG and TC concentrations, AI index, as well as level of HDL and HDL/TC ratio were not significantly affected by the supplementation of the diet with CBEs. These results are consistent with that of other studies which have revealed that cocoa powder and the cocoa phenolic extracts have favorable anti-inflammatory and hypolipidemic effects [7,34,35,38,73]. Previous studies with animal models have demonstrated that supplementation with cocoa polyphenols can favorably reduce the increased TG level in animals fed a high fat diet [73]. It is possible that the presence of cocoa phenolics, methylxanthines, and fibers prevents the treated rats from developing obesity by the decrease of the dietary cholesterol absorption in the intestinal tract or interference in the biosynthesis of cholesterol [7,34,35,38,71,73]. The applied experimental diets also did not significantly influence the activity of transaminases (ALT and AST) indicative of liver functioning and the content of calcium, phosphorus, and magnesium in the rats’ blood serum.

## 4. Conclusions

This study demonstrated that the presence of raw and roasted CBEs in the diets had only a slight effect on the growth parameters and activity of the enzymes of the cecal microflora of rats, but it caused a marked increase in the blood antioxidant capacity. Furthermore, the raw CBE in rats fed the high-fat diet showed a significant ability to inhibit lipid peroxidation in heart and more effectively increases hepatic GSH concentrations, with the latter resulting in a desirable increase in the GSH/GSSG ratio. The dietary treatments with raw cocoa beans preparations resulted in non-significant tendency in serum lipid profile in rats fed with the high-fat diet. Results of this study contribute to the evidence that dietary supplementation with raw and roasted CBEs might exert health promoting effects. Nevertheless, the metabolism of polyphenols is different between animal models and humans. Therefore, further studies are needed to confirm the effect of CBE supplementation on the physiological function of human.

## Figures and Tables

**Table 1:** Composition of cocoa bean extracts.

Compound (mg/mg DW)	Raw CBE	Roasted CBE
***Flavan-3-ols***		
(+)-Catechin	0.24 ± 0.01^b^	0.47 ± 0.02^a^
(−)-Epicatechin	8.68 ± 0.04^b^	6.35 ± 0.03^a^
Epigallocatechin	1.36 ± 0.01^b^	0.34 ± 0.01^a^
Procyanidin B2	7.59 ± 0.02^b^	7.21 ± 0.02^a^
Procyanidin C1	2.44 ± 0.02^b^	1.36 ± 0.01^a^
Other procyanidins	11.4 0 ± 0.05^b^	10.41 ± 0.04^a^
***Flavonols***		
Quercetin	0.01 ± 0.00^a^	***n***d
Quercetin 3-***O***-glucoside	0.30 ± 0.01^a^	0.26 ± 0.01^a^
Quercetin 3-***O***-arabinoside	0.31 ± 0.01^b^	0.27 ± 0.01^a^
Quercetin 3-***O***-galactoside	0.09 ± 0.01^a^	0.08 ± 0.00^a^
***Phenolic acids***		
Gallic acid	***n***d	1.85 ± 0.02^a^
Total phenolics	32.48 ± 0.18^b^	28.62 ± 0.17^a^
***Methylxanthines***		
Theobromine	57.56 ± 0.23	57.69 ± 0.19
Caffeine	5.03 ± 0.12^a^	5.65 ± 0.10^b^
Total methylxanthines	62.59± 0.35^a^	63.34± 0.29^b^

Mean values not sharing the same superscript letters (a-b) within a row are significantly different at *p* < 0.05; *n*d—not detected.

**Table 2:** Composition of the group-specific diets.

(%)	Diet^1^			
	D_CS_	D_CF_	D_RW_	D_RT_
Casein	20.0	20.0	20.0	20.0
DL-methionine	0.3	0.3	0.3	0.3
Rapeseed oil	7.0	7.0	7.0	7.0
Palm oil	-	14.0	14.0	14.0
Cellulose	5.0	2.0	2.0	2.0
Sucrose	10.0	10.0	10.0	10.0
Mineral blend	3.5	3.5	3.5	3.5
Vitamin blend	1.0	1.0	1.0	1.0
Choline chloride	0.2	0.2	0.2	0.2
Raw cocoa beans extract	-	-	2.0	-
Roasted cocoa beans extract	-	-	-	2.0
Corn starch	53.0	42.0	40.0	40.0

^1^Diet: D_CS_—control standard diet, D_CF_—control high-fat diet, D_RW_—diet enriched with freeze-dried raw cocoa beans extract, D_RT_—diet enriched with freeze-dried roasted cocoa beans extract.

**Table 3:** Dietary intake, body weight gain, and body fat/lean tissue of rats fed experimental diets. Data are expressed as mean ±SEM, *n* = 7.

	Diet				*P*-Value
	D_CS_	D_CF_	D_RW_	D_RT_	
Dietary intake (g/day/rat)	19.2 ± 0.5^b^	16.7 ± 0.5^a^	17.1 ± 0.4^a^	16.8± 0.3^a^	<0.001
Food energy intake (kJ)	326.5 ± 8.5^a^	337.2 ± 10.1^c^	339.5 ± 7.9^c^	333.5 ± 6.0^b^	0.045
Initial weight (g)	166.0 ± 0.4	165.9 ± 0.5	165.7 ± 0.2	165.8 ± 0.5	0.854
Final weight (g)	224.8 ± 0.5	234.1 ± 0.4	231.9 ± 0.6	232.6 ± 0.2	0.099
BWG (%)	35.4 ± 0.7	41.2 ± 0.8	39.9 ± 0.8	40.3 ± 0.6	0.101
Fat tissue (% BW)	31.2 ± 0.1	32.6 ± 0.1	32.5 ± 0.2	32.4 ± 0.1	0.091
Lean tissue (% BW)	42.4 ± 0.1	40.7 ± 0.1	41.6 ± 0.1	41.4 ± 0.1	0.212

Diet: D_CS_—control standard diet, D_CF_—control high-fat diet, D_RW_—diet enriched with freeze-dried raw cocoa beans extract, D_RT_—diet enriched with freeze-dried roasted cocoa beans extract. BWG—body weight gain. BW—body weight. SEM: standard error of the mean. Mean values not sharing the same superscript letters (a-c) within a row are significantly different at *p* < 0.05.

**Table 4:** Parameters of the small intestine, cecum, and colon of rats fed experimental diets. Data are expressed as mean ±SEM, n = 7.

	Diet				*p*-Value
	D_CS_	D_CF_	D_RW_	D_RT_	
***Small intestine***					
Tissue with content^1^	2.30 ± 0.08	2.39 ± 0.06	2.39 ± 0.11	2.27 ± 0.09	0.492
Content pH	6.84 ± 0.07	6.59 ± 0.04	6.88 ± 0.03	6.82 ± 0.06	0.189
Saccharase activity^2^	5.02 ± 0.09^c^	2.79 ± 0.06^a^	3.78 ± 0.17^a,b^	3.23 ± 0.15^a^	0.006
Maltase activity^2^	26.9a ± 0.5^c^	19.3^b^ ± 0.8^a^	23.9 ± 0.3^b^	19.7^b^ ± 0.6^a^	0.026
Lactase activity^2^	1.24 ± 0.09^c^	0.74 ± 0.07^a^	0.90 ± 0.04^b^	0.90 ± 0.05^b^	0.036
***Cecum***					
Tissue^1^	0.220 ± 0.019	0.211 ± 0.024	0.219 ± 0.021	0.216 ± 0.015	0.665
Content^1^	0.182 ± 0.017	0.222 ± 0.008	0.200 ± 0.012	0.269 ± 0.009	0.094
DM (%)	24.9 ± 0.5^b,c^	25.9 ± 0.3 ^d^	22.3 ± 0.4^a^	23.6 ± 0.2^a,b^	0.003
NH_3_ (mg/g)	0.254 ± 0.009	0.289 ± 0.017	0.257 ± 0.011	0.263 ± 0.008	0.246
Content pH	7.18 ± 0.08	7.13 ± 0.05	7.14 ± 0.04	7.12 ± 0.05	0.516
***Colon***					
Tissue^1^	0.350 ± 0.020^b^	0.303 ± 0.018^a^	0.296 ± 0.024^a^	0.298 ± 0.013^a^	0.019
Content^1^	0.149 ± 0.019^a^	0.135 ± 0.022^a^	0.209 ± 0.030^c^	0.160 ± 0.021^b^	0.010
Content pH	6.58 ± 0.09^a^	6.54 ± 0.08^a^	6.60 ± 0.03^a^	6.43 ± 0.05^a^	0.165

Diet: D_CS_—control standard diet, D_CF_—control high-fat diet, D_RW_—diet enriched with freeze-dried raw cocoa beans extract, D_RT_—diet enriched with freeze-dried roasted cocoa beans extract. SEM: standard error of the mean, DM: Dry matter of digesta (%). Mean values not sharing the same superscript letters (a-d) within a row are significantly different at *p* < 0.05. ^1^mass with contents, g/100 g BW; ^2^ µmol/min/g protein; BW, body weight.

**Table 5:** Volatile fatty acids (VFAs) in the rats’ cecum content. Data are expressed as mean ±SEM, *n* = 7.

	Diet				p-Value
	D_CS_	D_CF_	D_RW_	D_RT_	
VFA (µmol/g content)					
Acetic	50.6 ± 0.2^a,b^	50.3 ± 0.1^b^	58.5 ± 0.1^a,b^	70.7 ± 0.1^a^	0.029
Propionic	17.9 ± 0.4^a,b^	18.2 ± 0.1^a,b^	16.2 ± 0.1^b^	21.7 ± 0.1^a^	0.023
Iso-butyric	2.73 ± 0.05	2.90 ± 0.04	2.79 ± 0.03	2.59 ± 0.05	0.278
Butyric	7.81 ± 0.2^a,b^	5.28 ± 0.1^b^	9.18 ± 0.3^a,b^	10.4 ± 0.1^a^	0.016
Iso-valeric	1.42 ± 0.11^b^	1.81 ± 0.09^a^	1.67 ± 0.08^a,b^	1.58 ± 0.10^,b^	0.026
Valeric	1.34 ± 0.08	1.69 ± 0.07	1.34 ± 0.06	1.74 ± 0.99	0.065
Iso-butyric, iso-valeric, valeric	5.49 ± 0.02	6.40 ± 0.06	5.80 ± 0.02	5.91 ± 0.04	0.082
VFA total	89.8 ± 0.4^a,b^	80.2 ± 0.3^b^	89.7 ± 0.4^a,b^	109.0 ± 0.6^a^	0.026
VFA pool (µmol/100 g BW)					
Acetic	10.5 ± 0.1^b^	11.0 ± 0.3^b^	11.4 ± 0.2^b^	18.4 ± 0.2^a^	0.008
Propionic	3.13 ± 0.01^b^	3.96 ± 0.04^b^	3.22 ± 0.021^b^	5.71 ± 0.07^a^	0.004
Butyric	1.32 ± 0.08^b^	1.19 ± 0.05^b^	1.97 ± 0.04^a,b^	2.75 ± 0.08^a^	0.036
Iso-butyric, iso-valeric, valeric	0.98 ± 0.02^b^	1.38 ± 0.01^a,b^	1.18 ± 0.01^a,b^	1.56 ± 0.02^a^	0.047
VFA total	15.9 ± 0.3^b^	17.5 ± 0.1^b^	17.8 ± 0.2^b^	21.4 ± 0.4^a^	0.008
VFA profile (% total)					
Acetic	65.1 ± 0.1	62.7 ± 0.2	64.5 ± 0.1	65.1 ± 0.5	0.360
Propionic	20.1 ± 0.2^a,b^	22.6 ± 0.3^a^	18.4 ± 0.3^b^	20.2 ± 0.4^a,b^	0.005
Butyric	50.6 ± 0.3^a,b^	50.3 ± 0.1^b^	58.5 ± 0.3^a,b^	70.7 ± 0.2^a^	0.020

Diet: D_CS_—control standard diet, D_CF_—control high-fat diet, D_RW_—diet enriched with freeze-dried raw cocoa beans extract, D_RT_—diet enriched with freeze-dried roasted cocoa beans extract. SEM: standard error of the mean. Mean values not sharing the same superscript letters (a-b) within a row are significantly different at *p* < 0.05.

**Table 6:** Cecal bacterial enzyme activity and their release rate into the intestinal environment in rats fed experimental diets. Data are expressed as mean ±SEM, *n* = 7.

	Diet				*p*-Value
	D_CS_	D_CF_	D_RW_	D_RT_	
***α-glucosidase***					
Extracellular^1^	19.1 ± 0.3	20.6 ± 0.2	15.2 ± 0.4	16.7 ± 0.3	0.109
Intracellular^1^	3.94 ± 0.09	2.99 ± 0.07	3.40 ± 0.08	3.40 ± 0.11	0.590
Total^1^	23.0 ± 0.7	23.7 ± 0.4	18.6 ± 0.5	20.0 ± 0.7	0.222
Release degree (%)	84.4 ± 0.9	86.8 ± 0.8	85.1 ± 0.8	82.9 ± 0.6	0.548
***β-glucosidase***					
Extracellular^1^	0.855±0.004^a^	0.865±0.007^a^	2.960±0.011^b^	3.400 ± 0.009^b^	<0.001
Intracellular^1^	1.64 ± 0.16	1.44 ± 0.15	2.15 ± 0.17	2.14 ± 0.16	0.540
Total^1^	2.49 ± 0.04^a^	2.30 ± 0.08^a^	5.11 ± 0.12^b^	5.54 ± 0.10^b^	0.026
Release degree (%)	47.9 ± 1.1	48.8 ± 0.9	65.1 ± 0.8	67.4 ± 1.2	0.255
***α-galactosidase***					
Extracellular^1^	4.73 ± 0.02^a^	4.98 ± 0.04^a^	10.70 ± 0.08^c^	6.74 ± 0.07^b^	0.003
Intracellular^1^	6.70 ± 0.04^b^	2.97 ± 0.02^a^	6.83 ± 0.05^b^	8.66 ± 0.09^c^	0.004
Total^1^	11.40 ± 0.03^b^	7.95 ± 0.04^a^	17.50 ± 0.03^c^	15.40 ± 0.05^c^	0.005
Release degree (%)	43.3 ± 0.2^a^	63.2 ± 0.3^b^	62.6 ± 0.2^b^	42.3 ± 0.1^a^	0.025
***β-galactosidase***					
Extracellular^1^	9.04 ± 0.09	13.30 ± 0.11	8.92 ± 0.09	8.63 ± 0.13	0.109
Intracellular^1^	4.47 ± 0.07^b^	5.81 ± 0.03^b^	4.31 ± 0.04^b^	1.61 ± 0.09^a^	0.003
Total^1^	13.5 ± 0.4^a,b^	19.1 ± 0.8^c^	13.2 ± 0.6^a,b^	10.2 ± 0.8^a^	0.017
Release degree (%)	67.9 ± 0.9^a^	69.6 ± 1.4^a^	64.9 ± 1.5^a^	83.9 ± 1.4^b^	0.008
***β-glucuronidase***					
Extracellular^1^	13.3 ± 0.3	17.2 ± 0.4	12.5 ± 0.2	12.0 ± 0.3	0.140
Intracellular^1^	14.90 ± 0.08^b^	11.80 ± 0.07^a,b^	10.30 ± 0.05^a,b^	6.96 ± 0.06^a^	0.003
Total^1^	28.1± 0.5	29.0 ± 0.4	22. 8± 0.6	18.9 ± 0.2	0.060
Release degree (%)	48.2 ± 0.6^b^	58.3 ± 0.7^a,b^	51.4 ± 0.5^a,b^	64.1 ± 0.3^a^	0.024
Total^1^	23.0 ± 0.9	23.7 ± 0.8	18.6 ± 1.3	20.0 ± 0.9	0.109

Diet: D_CS_—control standard diet, D_CF_—control high-fat diet, D_RW_—diet enriched with freeze-dried raw cocoa beans extract, D_RT_—diet enriched with freeze-dried roasted cocoa beans extract. SEM: standard error of the mean. Mean values not sharing the same superscript letters (a-c) within a row are significantly different at *p* < 0.05. ^1^ µmol/h/g content; Release rate, extracellular expressed as percent of total activity.

**Table 7:** Antioxidant status and TBARS levels of the liver, kidneys, and heart in rats fed experimental diets. Data are expressed as mean ±SEM, *n* = 7.

	Diet				*p*-Value
	D_CS_	D_CF_	D_RW_	D_RT_	
***Liver***					
Mass (g/100 g BW)	3.47 ± 0.09	3.42 ± 0.04	3.30 ± 0.05	3.38 ± 0.07	0.686
TBARS	859 ± 12	967 ± 15	839 ± 19	885 ± 21	0.084
GSH+GSSG (μmol/g)	23.20 ± 0.04^b^	8.93 ± 0.05^a^	21.70 ± 0.06^b^	19.50 ± 0.07^b^	<0.001
GSH (μmol/g)	17.00 ± 0.06^b^	3.33 ± 0.03^a^	14.80 ± 0.05^b^	12.90 ± 0.08^b^	<0.001
GSSG (μmol/g)	6.25 ± 0.04	5.60 ± 0.03	6.92 ± 0.07	6.59 ± 0.04	0.075
GSH/GSSG	2.65 ± 0.09^b^	0.61 ± 0.04^a^	2.13 ± 0.08^b^	1.92 ± 0.07^b^	<0.001
***Kidneys***					
Mass (g/100 g BW)	0.710 ± 0.006	0.698 ± 0.004	0.700 ± 0.005	0.716 ± 0.003	0.644
TBARS	1379 ± 23	1534 ± 19	1424 ± 21	1492 ± 24	0.466
***Heart***					
Mass (g/100 g BW)	0.305 ± 0.003	0.309 ± 0.005	0.308 ± 0.002	0.296 ± 0.003	0.483
TBARS	1011 ± 18^b^	1191 ± 24^c^	933 ± 20^a^	1028 ± 19^b^	0.023

Diet: D_CS_—control standard diet, D_CF_—control high-fat diet, D_RW_—diet enriched with freeze-dried raw cocoa beans extract, D_RT_—diet enriched with freeze-dried roasted cocoa beans extract. TBARS: content of substances reacting with thiobarbituric acid, ng/g tissue. SEM: standard error of the mean. Mean values not sharing the same superscript letters (a-c) within a row are significantly different at *p* < 0.05.

**Table 8:** Selected biochemical characteristics of rats’ blood serum. Data are expressed as mean ± SEM, *n* = 7.

	Diet				p-Value
	D_CS_	D_CF_	D_RW_	D_RT_	
glucose (mmol/L)	7.41 ± 0.03^a^	7.60 ± 0.02^a^	8.95 ± 0.03^b^	8.83 ± 0.04^b^	0.020
TG (mmol/L)	0.91 ± 0.02	0.86 ± 0.03	0.92 ± 0.04	0.96 ± 0.03	0.583
TC (mmol/L)	1.65 ± 0.05	1.66 ± 0.05	1.62 ± 0.03	1.78 ± 0.02	0.283
HDL (mmol/L)	0.75 ± 0.04	0.69 ± 0.02	0.72 ± 0.01	0.79 ± 0.03	0.226
HDL profile (% TC)	45.2 ± 0.9	41.6 ± 0.7	45.2 ± 0.5	44.3 ± 0.6	0.214
non-HDL (mmol/L)	0.90 ± 0.03	0.97 ± 0.03	0.90 ± 0.03	0.99 ± 0.02	0.354
AI	0.08 ± 0.02	0.10 ± 0.04	0.06 ± 0.01	0.07 ± 0.03	0.689
AII	1.21 ± 0.05	1.45 ± 0.07	1.24 ± 0.06	1.29 ± 0.04	0.158
uric acid (μmol/L)	52.4 ± 1.5	31.4 ± 1.0	36.7 ± 1.6	37.9 ± 1.2	0.054
creatinine (μmol/L)	13.9 ± 0.9	13.5 ± 0.5	16.0 ± 0.7	10.7 ± 0.8	0.055
urea (mmol/L)	4.94 ± 0.04	4.30 ± 0.06	4.85 ± 0.08	4.36 ± 0.05	0.222
AST (U/L)	67.8 ± 1.7	70.0 ± 1.9	69.1 ± 1.8	66.2 ± 1.5	0.523
ALT (U/L)	19.0 ± 1.2	20.0 ± 0.7	21.8 ± 1.1	21.3 ± 0.9	0.312
calcium (mmol/L)	2.57 ± 0.04	2.50 ± 0.01	2.56 ± 0.03	2.56 ± 0.04	0.212
phosphorus (mmol/L)	2.80 ± 0.07	2.78 ± 0.06	2.77 ± 0.05	2.75 ± 0.05	0.780
magnesium (mmol/L)	0.84 ± 0.04	0.86 ± 0.02	0.85 ± 0.01	0.85 ± 0.04	0.484

Diet: D_CS_—control standard diet, D_CF_—control high-fat diet, D_RW_—diet enriched with freeze-dried raw cocoa beans extract, D_RT_—diet enriched with freeze-dried roasted cocoa beans extract. TG—triacylglycerols; TC—total cholesterol; HDL—high-density lipoprotein; AI—atherogenicity index of the diet I (log(TG/HDL)); AII—atherogenicity index of the diet II ((TC-HDL)/HDL); AST—aspartate transaminase; ALT—alanine transaminase. SEM: standard error of the mean. Mean values not sharing the same superscript letters (a-b) within a row are significantly different at *p* < 0.05.
